# Age and sex as risk factors for health-related quality of life outcomes in patients with glioma: a CODAGLIO 2.0 analysis

**DOI:** 10.1093/oncolo/oyag005

**Published:** 2026-01-22

**Authors:** Ogechukwu A Asogwa, Linda Dirven, Neil K Aaronson, Brigitta G Baumert, Martin van den Bent, Alba A Brandes, Paul M Clement, Corneel Coens, Olivier Chinot, Thierry Gorlia, Ulrich Herrlinger, Caroline Hertler, Florence Keime-Guibert, Emilie Le Rhun, Luigi Lim, Annika Malmström, Christine Marosi, Francesca Martinelli, Matthijs van der Meulen, Kathy Oliver, Andrea Pace, Claudia Panciroli, Jaap C Reijneveld, Mirjam Renovanz, Patrick Roth, Clemens Seidel, Roger Stupp, Wolfgang Wick, Michael Weller, Martin J B Taphoorn, Johan A F Koekkoek, EORTC Quality of Life Group and the EORTC Brain Tumor Group

**Affiliations:** Department of Neurology, Leiden University Medical Center, ZA 2333, Leiden, The Netherlands; Department of Neurology, Leiden University Medical Center, ZA 2333, Leiden, The Netherlands; Division of Psychosocial Research and Epidemiology, The Netherlands Cancer Institute, Postbus 90203 1006 BE Amsterdam, The Netherlands; Institute of Radiation-Oncology, Cantonal Hospital Graubünden, 7000 Chur, Switzerland; The Brain Tumor Center at Erasmus MC Cancer Institute, Rotterdam 3015GD, The Netherlands; Department of Medical Oncology, AUSL-IRCCS Scienze Neurologiche, Bologna 40124, Italy; Department of Oncology, KU Leuven, Leuven 3000, Belgium; European Organisation for Research and Treatment of Cancer, Brussels 1200, Belgium; Department of Neuro-Oncology, Aix-Marseille Université, CHU Timone, AP-HM, Marseille, France; European Organisation for Research and Treatment of Cancer, Brussels 1200, Belgium; Department of Neurooncology, Center for Neurology and Center for Integrated Oncology, University of Bonn Medical Center, Bonn, Germany; Department of Neurology, University Hospital and University of Zurich, Zurich 8091, Switzerland; Competence Center Palliative Care, Department of Radiation Oncology (ii), University Hospital and University of Zurich, Zürich 8091, Switzerland; Institut de Recherche International Servier 22, Gif sur Yvette 91190, France; Department of Neurosurgery, Clinical Neuroscience Center, University Hospital and University of Zurich, Zurich 8091, Switzerland; European Organisation for Research and Treatment of Cancer, Brussels 1200, Belgium; Clinical Department of Geriatrics and Palliative Medicine in Linköping, 581 85, Linköping, Sweden; Department of Medicine I, Division of Oncology, Medical University of Vienna, Vienna 1090, Austria; European Organisation for Research and Treatment of Cancer, Brussels 1200, Belgium; Department of Neurology, Leiden University Medical Center, ZA 2333, Leiden, The Netherlands; International Brain Tumour Alliance, Tadworth KT20 5WQ, United Kingdom; IRCCS Regina Elena Cancer Institute, Neuro-Oncology Unit, Rome 00144, Italy; Clinical Investigation Unit—Institut Català d’Oncologia, Badalona 08916, Spain. Neurology Unit—I.R.C.C.S. Ospedale San Raffaele, Milan 20132, Italy; Department of Neurology and Brain Tumour Center Amsterdam, Amsterdam University Medical Center, Amsterdam 1105, The Netherlands; Department of Neurology and Interdisciplinary Neuro-Oncology, Hertie Institute for Clinical Brain Research, Eberhard Karls University, Tübingen 72074, Germany; Center for Neuro-Oncology, Comprehensive Cancer Center Tübingen-Stuttgart, University Hospital Tübingen, Tubingen 72076, Germany; Department of Neurology, University Hospital and University of Zurich, Zurich 8091, Switzerland; Department of Radiation Oncology, University of Leipzig Medical Center, Leipzig 04103, Germany; Departments of Oncology and Neurosurgery, Centre Hospitalier Universitaire Vaudois (CHUV) and University of Lausanne, Lausanne CH-1015, Switzerland; Malnati Brain Tumor Institute, Lurie Comprehensive Cancer Center, Northwestern University, Chicago, IL 60611, United States; Neurology Clinic and European Centre for Neurooncology (EZN), University Hospital Heideberg, Heidelberg 69120, Germany; German Consortium of Translational Cancer Research, Clinical Cooperation Unit Neuro-oncology, German Cancer Research Center, Heidelberg 69120, Germany; Department of Neurology, University Hospital and University of Zurich, Zurich 8091, Switzerland; Department of Neurology, Leiden University Medical Center, ZA 2333, Leiden, The Netherlands; Department of Neurology, Leiden University Medical Center, ZA 2333, Leiden, The Netherlands

**Keywords:** brain tumor, health-related quality of life (HRQoL), age, sex, general population normative data

## Abstract

**Background:**

We assessed the clinical relevance of age and sex as risk factors for health-related quality of life (HRQoL) in patients with adult-type diffuse glioma.

**Materials and Methods:**

The CODAGLIO 2.0 database contains 16 randomized trials from 5369 patients with glioma. Patients’ HRQoL was assessed using EORTC QLQ-C30 and QLQ-BN20 questionnaires. In 8 HRQoL scales, we compared mean HRQoL at baseline with the general population and evaluated factors associated with HRQoL over time using linear mixed models (LMMs). We used the anchor-based minimally important difference to interpret clinically relevant changes.

**Results:**

We included 4301 patients with baseline HRQoL followed up to 3 months. Compared to the general population, patients with glioma at baseline had statistically and clinically relevant worse HRQoL, which was still evident after stratifying by age and sex groups. In LMMs, compared to patients aged ≤60 years, those >60 years had statistically significant associations with worse physical functioning: −2.40 (95% confidence interval [CI] −4.14 to −0.71), better social: 4.88 (2.68-7.30) and role: 3.79 (1.39-6.16) functioning, and less fatigue: −3.43 (−5.44 to −1.33) and pain: −4.56 (−6.18 to −2.93). Compared to men, women had statistically significant associations with worse physical and social functioning and more fatigue and pain. Associations between age, sex, and HRQoL were not clinically relevant. Performance status had clinically relevant associations in 5/8 scales.

**Conclusion:**

Patients with glioma have clinically relevant worse HRQoL compared to the general population. There are statistically but not clinically significant associations between age, sex, and certain HRQoL scales.

Implications for Practice This study demonstrates that although age and sex are statistically associated with some health-related quality of life (HRQoL) domains in patients with glioma, these associations are not clinically meaningful. In contrast, WHO performance status is strongly and clinically relevantly associated with HRQoL. For clinical practice, this underlines the importance of prioritizing performance status in decision-making and supportive care planning. Clinicians should be aware that glioma patients experience significant HRQoL impairments compared to the general population, highlighting the need for early, individualized supportive interventions to maintain or improve functioning and HRQoL.

## Introduction

Gliomas are the most frequent primary malignant neoplasms of the central nervous system (CNS)^1^ in adults, with an age-standardized incidence rate of 8.6 per 100 000 person-years.^2^ Despite advances in multi–modal therapy, including surgery, radiotherapy, and chemotherapy, patients with glioma will still experience an increased burden of symptoms,^3^^,^^4^ (multi)morbidity,^5^^,^^6^ and poor survival.^2^ Depending on the tumor type, the median survival time ranges from 9 to 21 months for patients with glioblastoma (isocitrate dehydrogenase [IDH] wildtype, CNS WHO grade 4), or median survival rates of 1-11 years for patients with astrocytoma IDH mutant, and 8-18 years for patients with oligodendroglioma (IDH mutant, 1p/19q codeleted).^2^^,^^7^ The limited survival time and lack of cure for most adult gliomas, alongside patients’ reports of fatigue, insomnia, and difficulty concentrating and remembering things as among the most relevant issues,^3^ have contributed to the understanding that not just the quantity of life but also the maintenance or improvement of health-related quality of life (HRQoL) is important in these patients throughout their disease course.^8^

While glioma may affect all ages and both sexes, demographic variables are associated with the incidence of glioma subtypes and patient prognosis.^9–13^ For example, Tewari et al.^9^ demonstrated that women with lower-grade gliomas have better survival rates at 2 and 5 years after diagnosis (94% and 87%, respectively) than men (92% and 78%, respectively). Gittleman et al.^11^ showed that women with glioblastoma had longer survival than men (median overall survival: 20.1 versus 17.8 months, respectively), particularly evident in the older age group (>56 years). Moreover, older age has been associated with lower overall survival times in several studies.^12^^,^^13^

Whether there are differences in HRQoL trajectories between sexes and age groups in patients with gliomas is still debated. Few studies that have investigated the association between age and sex and HRQoL outcomes in patients with glioma were hampered by small sample sizes and the inability to use anchor-based minimal important differences (MIDs) to differentiate between clinically and statistically significant associations. Instead of anchor-based MIDs, a 10-point MID was used in most studies, and it is well-documented that a 10-point MID underestimates clinically relevant changes.^14^ Given these limitations, available evidence is conflicting.^8^^,^^15–18^ For instance, female sex and older age were independently associated with a deterioration in some HRQoL scales (global health status, physical functioning, and role functioning) in patients with gliomas over time,^8^ while no association was found in another study.^16^ Furthermore, studies in patients with cancer and survivors showed that women reported fewer symptoms, including fatigue, dyspnea, anxiety, and depression, than men, and HRQoL scores decreased with increasing age for physical functioning.^19–21^

We aimed to gain more insight into age and sex risk factors of HRQoL outcomes in patients diagnosed with glioma. Therefore, we assessed whether HRQoL outcomes at baseline are worse in patients with gliomas compared to the general population and which risk factors, including sex and age, were independently associated with HRQoL patterns over time.

## Materials and methods

### Study design and population

This study is a longitudinal analysis using data from 4301 patients with a histologically confirmed glioma included in the CODAGLIO (COmbining clinical trial DAtasets in GLIOma) 2.0 database. The CODAGLIO 2.0 is a database consisting of a total of 5,369 individual patient data harmonized from 16 phase II and III interventional randomized control trials (RCTs) performed in the European Organisation for Research and Treatment of Cancer (EORTC) or other academic research groups (Figure S1). Data from patients were eligible for this current analysis if the patients were adults at least 18 years of age, diagnosed with histologically confirmed newly diagnosed or recurrent glioma, enrolled in one of the selected RCTs, and had baseline HRQoL assessments in at least one of these 2 questionnaires: the EORTC Quality of Life Questionnaire C30 (QLQ-C30)^22^ and the QLQ–Brain Neoplasm 20 module (QLQ-BN20).^23^ The institutional review board of Leiden University Medical Center approved this study.

### Instruments

HRQoL was assessed using the well-established and validated EORTC QLQ-C30 version 3.0 and/or the EORTC QLQ-BN20 questionnaires. The EORTC QLQ-C30 consists of 30 items that comprise 5 functional scales, including physical, role, emotional, cognitive, and social functioning; a global health status/quality of life scale; 3 symptom scales, including fatigue, pain, and nausea and vomiting; and 6 single items, including dyspnea, appetite loss, insomnia, constipation, diarrhea, and financial difficulties. The EORTC QLQ-BN20 is a disease-specific questionnaire that supplements the EORTC QLQ-C30 and is developed for patients with a brain tumor. It contains 20 items that focus on symptoms specific to patients with brain tumors and their treatments. The questionnaire comprises 4 symptom scales (future uncertainty, visual disorder, motor dysfunction, and communication deficits) and 7 single items (headaches, seizures, drowsiness, hair loss, itchy skin, weakness of legs, and bladder control). All items are scored on a 4-point Likert scale ranging from not at all to very much, except for the global health status/quality of life scale, which is scored on a 7-point Likert scale ranging from very poor to excellent. Following the standard EORTC procedures,^24^ scores were transformed linearly to a 0-100 scale, with a higher score showing better functioning for the functional scales. In contrast, for the symptom scales, higher scores reflect increased symptom burden.

### Outcome variables

Following the recommendations of the Response Assessment in Neuro-Oncology (RANO) Fast Track Clinical Outcome Assessments (COA) Group,^25^ we selected 8 out of 26 HRQoL scales. These included symptoms such as pain, difficulty communicating, and seizures, and functioning scales including physical, cognitive, role, and social functioning. Additionally, fatigue was selected as an important and highly prevalent symptom in glioma patients.^26^

### Clinical, sociodemographic, and time variables

In addition to HRQoL outcomes, sociodemographic variables, including age and sex, and clinical variables, such as tumor type, disease stage, tumor progression, World Health Organisation Performance Status (WHO PS) score, prior resection, cancer treatments, antiseizure medications (ASM), and corticosteroid use, were also collected. HRQoL assessment times varied among the RCTs. Follow-up time points were defined as t0 and t1, representing 0 months, which was at baseline (time of randomization), and 3 months, respectively. Given the drop in the completeness of HRQoL questionnaires over time, linear mixed model (LMM) analyses were limited to only 3 months (t1), after which the availability of HRQoL data dropped below 75%.

## Statistical analysis

### Descriptive statistics

Patients’ sociodemographic and clinical characteristics, as well as HRQoL outcomes, were described as means with their standard deviations, medians with their interquartile ranges (IQRs), and numbers with percentages. Data were also examined for missing values, and the number of missing values was reported. Depending on the type of data, a chi-square test and an independent sample Mann–Whitney *U* test were used in assessing the differences in characteristics of patients with and without any HRQoL assessments.

### Comparison of HRQoL between glioma patients and the general population

Given that HRQoL data were not normally distributed, 10 000 bootstrap resampling with a percentile confidence interval (CI) were used to estimate HRQoL means and their 95% CI at baseline, stratified by sex and age groups (18-29, 30-39, 40-49, 50-59, 60-69, and ≥70 years). Mean differences (MD) in HRQoL scale scores in patients with glioma were compared with the European QLQ-C30 general population data, as published by Nolte et al.^27^ To identify whether statistical differences between the groups were clinically relevant, anchor-based MIDs were used.^28^ These are differences of 5 and −7 in physical functioning, 8 and −9 in role functioning, 5 and −6 in social functioning, −5.5 in cognitive functioning, 7.6 and −7.4 in fatigue, and 7 and −6 in pain, corresponding to increment and decrement, respectively. The anchor-based MIDs are used for clinical interpretation of QLQ-C30 results and mean the minimum change in HRQoL scores that is perceived as meaningful for patients and would inform a change in management. More information about MIDs was published previously.^28^ This analysis was only performed on prioritized EORTC QLQ-C30 scales, as there is no general population normative data for the EORTC QLQ-BN20 questionnaire.

### Association of age and sex with HRQoL outcomes

LMMs were used to evaluate which factors, including age and sex, were independently associated with the preselected HRQoL outcomes. Based on existing literature and expert opinion, relevant variables were selected and included in the multi–variable analyses. Multi–collinearity was assessed using the variance inflation factor and correlation matrix of fitted models. We performed analysis on each of the 8 HRQoL outcome variables. To meet model assumptions, we used the bootstrap approach to provide model estimates and the uncertainty of parameters. A CI excluding zero was deemed statistically significant in the multi–variable LMMs, while anchor-based MIDs were used to interpret changes that were clinically relevant.^28^ Beta coefficients of the model fit of 5 and −7 in physical functioning, 8 and −9 in role functioning, 5 and −6 in social functioning, −5.5 in cognitive functioning, 7.6 and −7.4 in fatigue, and 7 and −6 in pain, corresponding to increment and decrement, respectively, were considered clinically relevant. As anchor-based MIDs were only available for the EORTC QLQ-C30 questionnaire, clinically relevant anchor-based changes for communication deficits and seizures from the EORTC QLQ-BN20 could not be determined. For this reason, we used the standard 10-point MIDs for communication deficits and seizures.^29^ All analyses were performed with R version 4.3.1.

## Results

### Patient characteristics

**Figure 1.**
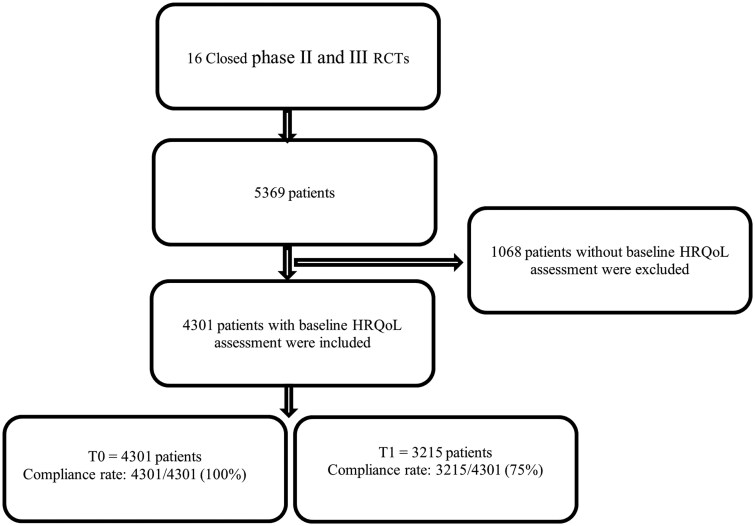
Flowchart showing the inclusion of patients in the study. Abbreviations: HRQoL, health-related quality of life; RCTs, randomized controlled trials; T0, baseline at 0 months; T1, follow-up at 3 months.

4301 (80%) out of 5369 patients in the database were eligible for this study because they had a baseline HRQoL assessment. Among the 4301 eligible patients, the compliance rate of their HRQoL assessment dropped to 75% (3215/4301) over a 3-month follow-up time. As a result of a huge drop (50%) in the compliance rate at t2 (6 months), LMM’s analysis was limited to only 3 months (t1) (Figure 1). In this group of 4301 eligible patients, 2638 (61%) were men, and the median age at diagnosis was 54 years (IQR 19). A total of 1615 (38%) had an excellent WHO PS (score 0), 2687 (63%) had undergone partial or complete resection, 2607 (61%) were diagnosed with glioblastoma, and 1449 (34%) had tumor progression within 3 months of follow-up. Except for sex, there were statistically significant differences in patients’ characteristics between patients with and without a baseline HRQoL assessment. Patients without baseline HRQoL assessment were older, not on ASMs, had worse WHO PS (≥1), were more often diagnosed with glioblastoma, and were more likely to be newly diagnosed patients as well (Table 1). Moreover, except for sex and disease status (newly vs. recurrent), there were also statistically significant differences in patients’ characteristics between patients with and without a 3-month follow-up HRQoL assessment. Patients without a 3-month follow-up HRQoL assessment were older, used more steroids, had worse WHO PS (≥1), and were more often diagnosed with glioblastoma (result not presented in a table).

**Table 1.** Sociodemographic and clinical characteristics of patients with and without baseline HRQoL assessment.

Characteristics	All patients, n (%)	Patients with baseline HRQoL assessment	Patients without baseline HRQoL assessment	2 sided Χ² p.value
Total number of participants	5369 (100.0)	4301 (80.1)	1068 (19.9)	
Sex				
*Men*	3237 (60.3)	2638 (61.3)	599 (56.1)	0.54
*Women*	2055 (38.3)	1661 (38.6)	394 (36.9)	
*Unknown*	77 (1.4)	2 (0.0)	75 (7.0)	
Age (years), median (IQR, min, max)	56 (20.0, 18.0, 92.0)	54.00 (19.0, 18.0, 84.0)	60.00 (21.0, 19.0, 84.0)	<0.01^a^
Age group				<0.01
*≤60*	3333 (62.1)	2937 (68.3)	396 (37.1)	
*>60*	1982 (36.9)	1364 (31.7)	618 (57.9)	
*Unknown*	54 (1.0)	0 (0.0)	54 (5.1)	
Age category				<0.01
*18-29*	247 (4.2)	226 (5.3)	21 (1.3)	
*30-39*	613 (10.3)	552 (12.8)	61 (3.7)	
*40-49*	982 (16.6)	863 (20.1)	119 (7.3)	
*50-59*	1341 (22.6)	1179 (27.4)	162 (9.9)	
*60-69*	1495 (25.2)	1060 (24.6)	435 (26.7)	
*≥70*	1199 (20.2)	421 (9.8)	778 (47.7)	
*Unknown*	54 (0.9)	0 (0.0)	54 (3.3)	
Disease status				<0.01
*Newly diagnosed*	4114 (76.6)	3177 (73.9)	937 (87.7)	
*First recurrence*	1255 (23.4)	1124 (26.1)	131 (12.3)	
Treatment				<0.01
*No*	35 (0.7)	0 (0.0)	35 (3.3)	
*ABT-414 alone*	86 (1.6)	61 (1.4)	25 (2.3)	
*Angio alone*	126 (2.3)	121 (2.8)	5 (0.5)	
*CT alone*	1086 (20.1)	867 (20.2)	219 (20.5)	
*CT and ABT-414*	88 (1.6)	66 (1.5)	22 (2.1)	
*CT and angio*	532 (9.9)	395 (9.2)	137 (12.8)	
*RT alone*	1416 (26.4)	1027 (23.9)	389 (36.4)	
*RT and angio*	49 (0.9)	46 (1.1)	3 (0.3)	
*RT and CT*	1529 (28.6)	1348 (31.3)	181 (16.9)	
*RT and CT and MRZ*	375 (7.0)	370 (8.6)	5 (0.5)	
*Unknown*	47 (0.9)	0 (0.0)	47 (4.4)	
Tumor type				<0.01
*IDHmutant glioma*	948 (17.7)	857 (19.9)	91 (8.5)	
*Glioblastoma* ^b^	3402 (63.4)	2607 (60.6)	795 (74.4)	
*Not otherwise specified* ^c^	1019 (19.0)	837 (19.5)	182 (17.0)	
Progression within 3 months follow-up				0.04
*No*	3261 (60.7)	2779 (64.6)	482 (45.1)	
*Yes*	1659 (30.9)	1449 (33.7)	210 (19.7)	
*Unknown*	449 (8.4)	73 (1.7)	376 (35.2)	
WHO PS				<0.01
*0*	1917 (35.7)	1615 (37.5)	302 (28.3)	
*≥1*	3376 (62.9)	2652 (61.7)	724 (67.8)	
*Unknown*	76 (1.4)	34 (0.8)	42 (3.9)	
Extent of resection				0.02
*Biopsy*	1365 (25.4)	1129 (26.2)	236 (22.1)	
*Partial or complete*	3371 (62.8)	2687 (62.5)	684 (64.0)	
*Unknown*	633 (11.8)	485 (11.3)	148 (13.9)	
Steroid use				<0.01
*No*	2184 (40.7)	1889 (43.9)	295 (27.6)	
*Yes*	2018 (37.6)	1667 (38.8)	351 (32.9)	
*Unknown*	1167 (21.7)	745 (17.3)	422 (39.5)	
Antiseizure medication				<0.01
*No*	819 (15.3)	501 (11.6)	318 (29.8)	
*Yes*	1312 (24.4)	1076 (25.0)	236 (22.1)	
*Unknown*	3238 (60.3)	2724 (63.3)	514 (48.1)	

Abbreviations: ABT-414, depatuxizumab mafodotin; Angio, Angiogenesis (bevacizumab); ASM, anti-seizure medication; CT, chemotherapy; Fup, follow-up; HRQoL, health related quality of life; IQR, interquartile range; max, maximum; Min, minimum; MRZ, Marizomib; N, numbers; RT, radiotherapy; WHO PS, World Health Organization Performance Status.

a Independent sample Mann–Whitney *U* test and Median test.

b Glioblastoma according to the WHO 2016 or 2021 classification of CNS tumors.

c Not otherwise specified: Patients with a diffuse glioma that cannot further be reclassified based on 2021 WHO criteria because of lacking information on IDH mutation and 1p19q codeletion status.

### Comparison of patients’ baseline HRQoL scale scores with the general population

**Figure 2.**
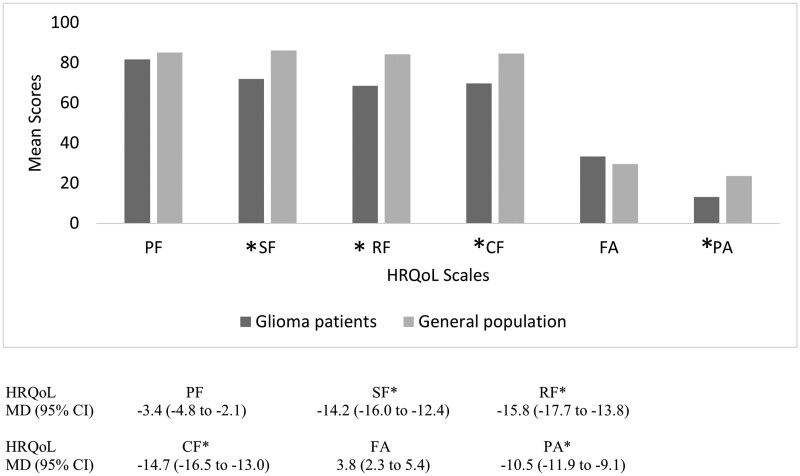
Overall mean score in the selected HRQoL scales at baseline in patients with glioma compared with the general population normative data. Abbreviations: CF, cognitive functioning; CI, confidence interval; FA, fatigue; MD, mean difference; PA, pain; PF, physical functioning; RF, role functioning; SF, social functioning. ^*^Both statistically and clinically relevant. A negative sign on the mean difference signifies a lower score in the patients with glioma compared to the general population, while a positive score means a higher score in the patients with glioma compared to the general population.

We compared the mean baseline scores of the selected HRQoL scales in 4301 patients with glioma with the general population normative data (Figure 2 and Table S1). Overall, there were statistically and clinically significant lower mean scores for social, role, and cognitive functioning in patients with glioma compared to the general population, with the MD varying between −14.2; 95% CI (−16.0 to −12.4) for social functioning and −15.8; 95% CI (−17.7 to −13.8) for role functioning. Interestingly, there was a statistically and clinically significant lower mean pain score in patients with glioma compared to the general population, with an MD of −10.5; 95% CI (−11.9 to −9.1). Although patients with glioma had statistically significantly worse physical functioning (−3.4; 95% CI [−4.8 to −2.1]) and more fatigue (3.8, 95% CI [2.3 to 5.4]) compared to the general population, these differences were not clinically relevant.

**Figure 3.**
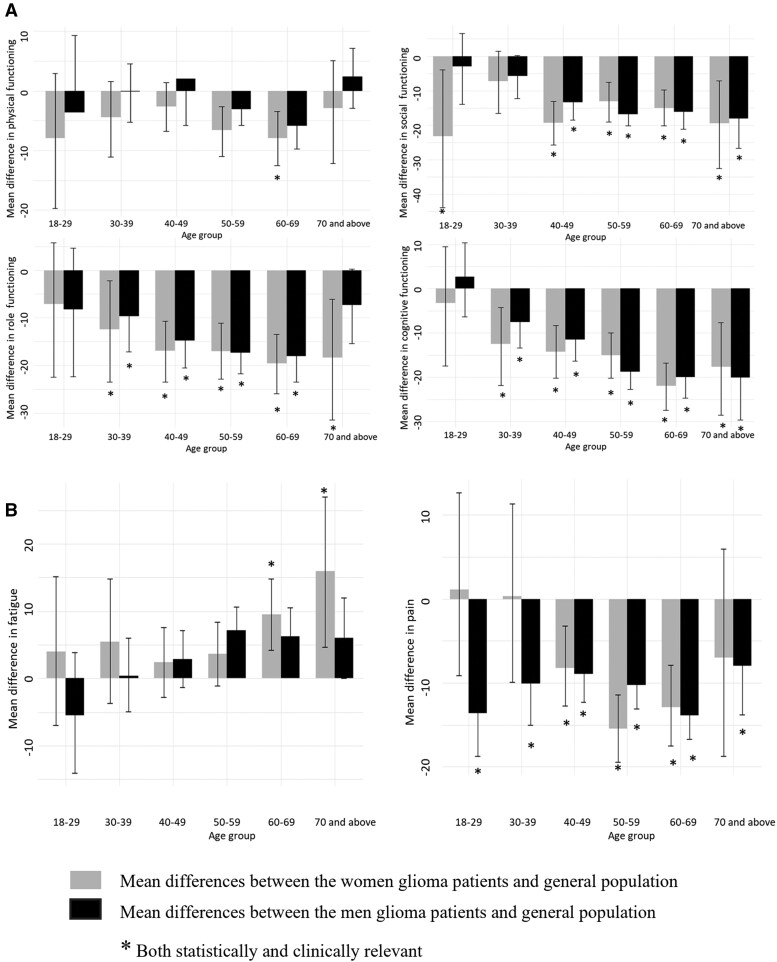
Mean differences between patients with glioma and the general population stratified by age and sex for selected functioning scales (A) and symptom scales (B). A negative sign on the mean difference signifies a lower score in patients with glioma compared to the general population, while a positive score means a higher score in patients with glioma compared to the general population.

After stratification by sex and age group (18-29, 30-39, 40-49, 50-59, 60-69, and ≥70 years) across the functioning scales, some subcategories of patients showed statistically and/or clinically significant differences at baseline compared to the general population normative data. For instance, compared to the general population, women with glioma aged 60-69 years had clinically relevant lower scores for physical functioning (MD is −7.9; 95% CI −12.5 to −3.4). For social functioning, there were clinically relevant lower scores in patients with glioma compared to the general population for women within 18-29 and ≥40 years old and men ≥40 years old (MD varies between −23.3 and −13.1 in women within 18-29 and 50-60 years old, respectively). Notably, there were clinically relevant lower scores for role (with the exception of men and women 18-29 years old and men above 70 years old) and cognitive functioning (with the exception of men and women 18-29 years old) in patients with glioma compared to the general population (MD varies between −7.5 in men 30-39 years old and −22.0 in women 60-69 years old). See Figure 3A and Table S1. Across the symptom scales, there was a clinically relevant higher fatigue score from age ≥60 in women with glioma compared to the general population (MD varies between 9.5 in men 60-69 years old and 15.9 in women ≥70 years old). On the contrary, clinically relevant lower pain scores were seen in patients with glioma compared to the general population for women between 40 and 69 years old and men of all age groups (MD varies between −8.0 in men ≥70 years old and −15.5 in women 50-59 years old). See Figure 3B and Table S1.

### Independent associations of age and sex with HRQoL outcomes

We estimated risk factors, including age and sex, that were independently associated with worse HRQoL outcomes over time (Table 2). Given the presence of multi–collinearity between variables in the model, anti-tumor treatment was excluded because it had multi–collinearity with disease status (newly diagnosed vs. recurrent tumor). Age and sex showed only a statistically significant association with certain HRQoL outcomes, but the association did not reach clinical relevance. Specifically, compared to patients ≤60 years, patients aged >60 years showed significantly worse physical functioning (−2.40; 95% CI: −4.14 to −0.71), better social functioning (4.88; 95% CI: 2.68 to 7.30) and role functioning (3.79; 95% CI: 1.39 to 6.16), and less fatigue (−3.43; 95% CI: −5.44 to −1.33) and less pain (−4.56; 95% CI: −6.18 to −2.93). No significant difference was seen for cognitive functioning, communication deficits, or seizures. Compared to men, women showed statistically significant association with worse physical functioning (−4.35; 95% CI: −5.73 to −2.86) and social functioning (−2.03; 95% CI: −3.98 to −0.06), more fatigue (4.90; 95% CI: 3.16 to 6.50), and more pain (3.37; 95% CI: 1.70 to 4.97), whereas no significant difference was seen for role functioning, cognitive functioning, communication deficits, or seizures. Additionally, compared to t0, patients had statistically significantly worse physical (−4.82; 95% CI: −5.73 to −3.98), role (−1.70; 95% CI: −3.05 to −0.34), and cognitive (−1.56; 95% CI: −2.63 to −0.53) functioning and higher fatigue (5.85; 95% CI: 4.68 to 7.06) and communication deficit (1.58; 95% CI: 0.70 to 2.57) scores at t1. Social functioning, pain, and seizures were not significant at t1 compared to t0 (Table 2). Similarly, other demographic and clinical variables were only statistically but non–clinically associated with HRQoL. Among all model variables, only WHO PS had both statistically and clinically significant associations with physical, role, social, and cognitive functioning, as well as fatigue, with beta coefficients ranging between −16.12 and 9.82 in role functioning and fatigue, respectively.

**Table 2.** Result of the linear mixed model analyses, showing the beta coefficients of the independent effect of age and sex on the selected functioning and symptom scales.

(A) Functioning scales	Physical functioning	Social functioning	Role functioning	Cognitive functioning	Fatigue	Pain	Communication	Seizures
	Beta coef (95% CI)	Beta coef (95% CI)	Beta coef (95% CI)	Beta coef (95% CI)	Beta coef (95% CI)	Beta coef (95% CI)	Beta coef (95% CI)	Beta coef (95% CI)
Age-group								
*≤60*	Ref	Ref	Ref	Ref	Ref	Ref	Ref	Ref
*>60*	−2.40 (−4.14 to −0.71)^a^	4.88 (2.68 to 7.30)^a^	3.79 (1.39 to 6.16)^a^	−0.74 (−2.78 to 1.25)	−3.43 (−5.44 to −1.33)^a^	−4.56 (−6.18 to −2.93)^a^	1.41 (−0.89 to 3.38)	−1.21 (−2.45 to 0.09)
Sex								
*Men*	Ref	Ref	Ref	Ref	Ref	Ref	Ref	Ref
*Women*	−4.35 (−5.73 to −2.86)^a^	−2.03 (−3.98 to −0.06)^a^	−1.20 (−3.20 to 0.83)	−0.78 (−2.53 to 1.01)	4.90 (3.16 to 6.50)^a^	3.37 (1.70 to 4.97)^a^	−0.32 (−2.08 to 1.48)	−0.51 (−1.75 to 0.71)
Follow-up time								
*T0*	Ref	Ref	Ref	Ref	Ref	Ref	Ref	Ref
*T1*	−4.82 (−5.73 to −3.98)^a^	−0.81 (−2.11 to 0.54)	−1.70 (−3.05 to −0.34)^a^	−1.56 (−2.63 to −0.53)^a^	5.85 (4.68 to 7.06)^a^	0.77 (−0.27 to 1.74)	1.58 (0.70 to 2.57)^a^	−0.50 (−1.44 to 0.34)
WHO PS								
*0*	Ref	Ref	Ref	Ref	Ref	Ref	Ref	Ref
*≥1*	−10.11 (−11.41 to −8.79)^b^	−10.68 (−12.60 to −8.80)^b^	−16.12 (−18.19 to −14.06)^b^	−10.46 (−12.15 to −8.81)^b^	9.82 (8.02 to 11.50)^b^	5.61 (4.07 to 7.12)^a^	8.39 (6.42 to 10.01)^a^	3.10 (1.89 to 4.41) ^a^
Extent of resection								
*Biopsy*	Ref	Ref	Ref	Ref	Ref	Ref	Ref	Ref
*Partial or Complete*	4.03 (1.86 to 6.29)^a^	0.16 (−2.73 to 2.81)	1.63 (−1.25 to 4.56)	0.52 (−2.14 to 3.14)	−2.13 (−4.70 to 0.41)	−0.62 (−2.75 to 1.58)	−1.95 (−4.63 to 0.85)	−5.51 (−7.76 to −3.31) ^a^
Steroid use								
*No*	Ref	Ref	Ref	Ref	Ref	Ref	Ref	Ref
*Yes*	−6.31 (−7.76 to −4.80)^a^	−4.87 (−7.03 to −2.79)^a^	−7.67 (−9.77 to −5.40)^a^	−4.71 (−6.58 to −2.92)^a^	4.96 (3.18 to 6.67)^a^	2.11 (0.55 to 3.64)^a^	3.39 (1.27 to 5.14)^a^	0.13 (−1.09 to 1.38)
Tumor status								
*Newly diagnosed*	Ref	Ref	Ref	Ref	Ref	Ref	Ref	Ref
*Recurrent*	−1.80 (−4.34 to 0.71)	1.58 (−1.68 to 4.77)	0.67 (−2.79 to 4.06)	−4.82 (−7.66 to −1.81)^a^	−1.38 (−4.39 to 1.57)	−0.64 (−3.30 to 1.75)	4.39 (1.06 to 7.53)^a^	−4.59 (−6.89 to −2.42)^a^
Tumor progression within 3-month follow-up								
*No*	Ref	Ref	Ref	Ref	Ref	Ref	Ref	Ref
*Yes*	−3.01 (−4.99 to −1.27)^a^	−3.22 (−5.45 to −0.80)^a^	−6.01 (−8.41 to −3.61)^a^	−3.87 (−6.20 to −1.70)^a^	3.12 (1.05 to 5.29)^a^	1.65 (−0.19 to 3.44)	4.18 (2.14 to 6.37)^a^	1.79 (0.58 to 3.15)^a^
Tumor type								
Other glioma	Ref	Ref	Ref	Ref	Ref	Ref	Ref	Ref
Glioblastoma	2.94 (1.23 to 4.79)^a^	1.90 (−0.76 to 4.48)	1.95 (−0.91 to 4.68)	4.58 (2.13 to 6.95)^a^	−1.80 (−4.17 to 0.55)	−3.28 (−5.35 to −1.26)^a^	−6.04 (−8.26 to −3.67)^a^	−5.42 (−7.39 to −3.58)^a^

Abbreviations: CI, confidence interval; Coef, coefficient; Ref, reference category; T0, baseline at 0 month; T1, follow-up at 3 months; WHO PS, World Health Organization Performance Status.

a Only statistically significant

b Both statistically and clinically significant.

## Discussion

Our study indicates that, at baseline, patients with glioma have both clinically and statistically significantly poorer HRQoL in the domains of social, role, and cognitive functioning, but also lower levels of pain compared to the general population. After stratification by sex and age groups, we found differences across certain age groups and sexes in patients compared to the general populations. For example, women with glioma aged ≥60 had a clinically relevant higher fatigue score compared to the general population. Moreover, in multi–variable analysis, age >60 compared to ≤60 years was statistically associated with worse physical functioning but better social and role functioning and less fatigue and pain. Women, compared to men, were significantly associated with worse physical and social functioning and more fatigue and pain. However, none of these differences in sexes (women vs. men) and age groups (60 vs. >60) met the criterion to be clinically relevant.

In line with other, but smaller, studies in patients with brain tumors,^30–34^ patients with glioma showed clinically relevant worse scores in social, role, and cognitive functioning compared to the general population at baseline, emphasizing the possibly severe impact of the tumor, extent of surgical resections, and previous antitumor treatment in patients with recurrent disease on HRQoL outcomes. Notably, the increasingly worse scores in social, role, and cognitive functioning and fatigue in both glioma and the general population with increasing age underline the increased age-related frailty, impacting daily functioning. On the contrary, similar to the findings by Osoba et al.^34^ and Klein et al.,^35^ our results show that pain scores were lower in patients with glioma at baseline. Nevertheless, fatigue is among the most noted prevalent symptoms in patients with a brain tumor at both pre- and post–treatment.^36^^,^^37^ In our study, compared to the general population, a clinically relevant worse fatigue score was observed at baseline only in women ≥60 years old, which further underscores the general frailty of old age in women. The clinically relevant worse fatigue scores in ≥60-year-old women could be of a multi–faceted origin, including a possible combined effect of hormonal and postmenopausal changes,^38^ as well as the effect of psychosocial factors (higher anxiety and depression in women compared to men).^17^ Importantly, given that symptomatology and levels of functioning differ between patients with glioma and the general population, this finding calls for a comprehensive assessment of patients’ symptoms and functioning at diagnosis to offer timely supportive care^39^ that would improve HRQoL in varying age and sex groups.

Previous studies in patients with brain tumors found a statistically significant association between age and HRQoL in a non–longitudinal analysis.^15^^,^^18^^,^^33^^,^^40–42^ Our study, after correcting for clinical variables in a longitudinal analysis, confirms that older age is associated with worse physical functioning.^15^^,^^18^^,^^40^^,^^42^ Additionally, our study also shows that older age is statistically associated with better social functioning and role functioning, but less fatigue and pain.^15^^,^^18^ Importantly, these associations do not reach clinical relevance. These findings might be explained by multiple other factors. The interaction between the complex process of aging and antitumor treatments might reduce physical functioning. For instance, the body undergoes physiological changes, including cellular damage and changes in brain function, during aging. At the same point, older patients treated with multi–modal therapy might have a lower capacity to tolerate these aggressive treatments compared to younger patients,^4^^,^^43^ mainly due to the presence of comorbidity and cellular damage, influencing patients’ vulnerability to treatment toxicity such as decreased daily functioning.^44^ Moreover, the association of older age with better social functioning and role functioning and less fatigue and pain compared to younger patients could also be explained by coping mechanisms and adjustments due to life experience. Older patients are likely to be retired or close to retirement and have a reduced workload as compared with younger patients^45^; they may have better coping and adaptation mechanisms, as well as more family and community support during the course of their disease, thereby reporting social functioning, role functioning, fatigue, and pain differently from the younger age groups.^45–47^

There are a few studies of sex differences in HRQoL in patients with brain tumors.^15^^,^^17^^,^^18^^,^^41^^,^^48^^,^^49^ These available studies showed mixed findings, and some did not focus on specific scales; rather, results were presented as total HRQoL and global health status score.^15^^,^^17^^,^^41^ Our results (worse physical and social functioning and more fatigue and pain in women than men) are consistent with those of Rogers et al.,^15^ as they show that women are associated with worse functioning status, energy level, and pain.^15^ However, other studies did not find any association with the analyzed HRQoL scales.^21^^,^^42^^,^^51^ Reasons for this difference may be attributed to the analytical methods comprising the use of Spearman’s rank correlation and non–longitudinal linear regression.^18^^,^^40^^,^^48^ Although the results of Rogers et al.^15^ were partial correlation coefficients adjusted for age and time since diagnosis, the consistent findings with ours underscore the need to initiate rehabilitation and supportive interventions targeting body strength, functioning, fatigue, and pain symptoms for the vulnerable women. Given mixed findings, further research is needed to understand the role of sex in HRQoL outcomes to necessitate appropriate tailoring of supportive therapy for these vulnerable affected subgroups in patients. Apart from the population of patients with brain tumors, other populations, such as the general and other cancer populations, reported similar findings to ours on worse physical functioning, and more pain and fatigue in women compared to men,^19^^,^^50^ but contradictory findings on other HRQoL scales.^19^^,^^20^^,^^50^ Notably, the cause of sex differences in HRQoL outcomes is still debated. However, besides the function of sex hormones in controlling muscle function,^38^ and the presence of psychological distress^17^ and multiple chronic illnesses that are more prevalent in women than in men might give insight into differences seen in HRQoL outcomes between the sexes in the general population^5^ which can be extrapolated to patients with glioma.

Although statistically significant, there appeared to be no clinically relevant independent association between age or sex and HRQoL outcomes. However, a strong clinically relevant association was found between lower performance status and worse HRQoL for 5 out of 8 preselected domains, implying that there is a need for clinicians to discuss treatment strategies with patients considering the performance status to obtain an acceptable balance between treatment efficacy and HRQoL. In addition, compared to the general population, HRQoL outcomes in patients with glioma, as seen in our study, are already worse in several domains at baseline, indicating that factors such as tumor progression and the side effects of anti-tumor treatments are not the sole determinants of lower HRQoL scores. Therefore, to maintain or improve HRQoL outcomes in patients with glioma, interventions aimed at reducing symptom burden and improving patients’ functioning are highly needed from the start of the disease.

The strength of this study lies in the large number of patients included in the study, ensuring reliable estimates. We used anchor-based MIDs to evaluate clinically relevant changes since the 10-point MID used in earlier research is very simplistic, underestimates clinically meaningful changes,^14^ and is unable to distinguish between domains and change direction.^51^ Moreover, considering the limited variables in the previous non–longitudinal studies, our longitudinal study included important variables to encourage the understanding of the interplay of diverse risk factors, thereby enabling the fostering of a comprehensive care plan for patients with glioma with a unique set of risks. Additionally, the normative reference data arise from 11 343 participants from specific countries and are assumed to be a good representation of the general population.^27^

Nevertheless, some limitations need to be considered. Due to the selection of participants, the generalizability of the results is limited. We included participants who were part of RCTs with stringent inclusion criteria; therefore, the study population does not fully capture the population seen in routine clinical practice, amounting to selection bias. Furthermore, patients who completed HRQoL assessments might represent a population with more favorable patient and tumor characteristics compared to patients without HRQoL assessments, contributing to additional selection bias. Among those who were included, compliance rates decreased over time, resulting in attrition bias. This problem is of particular relevance for patients with glioma, given their limited life expectancy, high risk of tumor progression, and progressive neurocognitive problems. Consequently, patients with better health conditions could be overrepresented over time. Finally, measurement bias could have been introduced due to irregular HRQoL assessments among the different trials.

## Conclusion

Our study highlights the complex interplay of age and sex as risk factors for HRQoL outcomes among patients with glioma and also the need to compare HRQoL not just within the brain tumor population but also to other population settings in order to comprehend the true burden of glioma and its treatment. Since worse HRQoL is indisputable in patients with glioma when compared to the general population, our study stresses the need for personalized interventions (supportive care and rehabilitation) that aim to reduce symptom burden and improve patients’ functioning. Given that age and sex were not independent clinically relevant risk factors for HRQoL, they should not be major determinants in clinical decision-making aimed at maintaining HRQoL. Instead, patient characteristics such as performance status appear to have greater relevance in impacting a patient’s HRQoL.

## Supplementary Material

oyag005_Supplementary_Data

## Data Availability

The data underlying this article will be shared on reasonable request to the corresponding author..
