# Supplementary material for: Age and sex as risk factors for health-related quality of life outcomes in patients with glioma: a CODAGLIO 2.0 analysis
Source: Oncologist. 2026 Jan 22;31(3):oyag005. doi: 10.1093/oncolo/oyag005 (PMC12948937; doi:10.1093/oncolo/oyag005)
Supplement: oyag005_Supplementary_Data [file oyag005_supplementary_data.zip › 20251113_Suplimentary Material (1).docx]

**Age and sex as risk factors for health-related quality of life outcomes in patients with glioma: a CODAGLIO 2.0 analysis**

**Supplementary Material**

Supplementary Figure S1: 16 closed phase II and III randomised control trials (RCTs)

**References for the primary publication of the 16 included trials**

1. NCT01443676 (ARTE): Wirsching HG, Tabatabai G, Roelcke U,et al. Bevacizumab plus hypofractionated radiotherapy versus radiotherapy alone in elderly patients with glioblastoma: the randomized, open-label, phase II ARTE trial. Ann Oncol. 2018 Jun 1;29(6):1423-1430.
2. NCT00430911: Keime-Guibert F, Chinot O, Taillandier L, et al.; Radiotherapy for glioblastoma in the elderly. N Engl J Med. 2007 Apr 12;356(15):1527-35.
3. NCT00941460 (DIRECTOR): Weller M, Tabatabai G, Kästner B, et al; DIRECTOR Study Group. MGMT Promoter Methylation Is a Strong Prognostic Biomarker for Benefit from Dose-Intensified Temozolomide Rechallenge in Progressive Glioblastoma: The DIRECTOR Trial. Clin Cancer Res. 2015 May 1;21(9):2057-64
4. ISRCTN45209900 (Neoadjuvant TMZ): Malmström A, Poulsen HS, Grønberg BH, et al. Postoperative neoadjuvant temozolomide before radiotherapy versus standard radiotherapy in patients 60 years or younger with anaplastic astrocytoma or glioblastoma: a randomized trial. Acta Oncol. 2017 Dec;56(12):1776-1785.
5. NTR1929 (BELOB): Taal W, Oosterkamp HM, Walenkamp AM, et al. Single-agent bevacizumab or lomustine versus a combination of bevacizumab plus lomustine in patients with recurrent glioblastoma (BELOB trial): a randomised controlled phase 2 trial. Lancet Oncol. 2014 Aug;15(9):943-53.
6. NCT01164189 (EORTC 26091 (TAVAREC)): van den Bent MJ, Klein M, Smits M, et al. Bevacizumab and temozolomide in patients with first recurrence of WHO grade II and III glioma, without 1p/19q co-deletion (TAVAREC): a randomised controlled phase 2 EORTC trial. Lancet Oncol. 2018 Sep;19(9):1170-1179.
7. NCT00967330 (Glarius): Herrlinger U, Schäfer N, Steinbach JP, et al. Bevacizumab Plus Irinotecan Versus Temozolomide in Newly Diagnosed O6-Methylguanine-DNA Methyltransferase Nonmethylated Glioblastoma: The Randomized GLARIUS Trial. J Clin Oncol. 2016 May 10;34(14):1611-9.
8. NCT02343406 (EORTC 1410 (INTELLANCE-2)): Van Den Bent M, Eoli M, Sepulveda JM, et al. INTELLANCE 2/EORTC 1410 randomized phase II study of Depatux-M alone and with temozolomide vs temozolomide or lomustine in recurrent EGFR amplified glioblastoma. Neuro Oncol. 2020 May 15;22(5):684-693.
9. ISRCTN81470623 (Nordic): Malmström A, Grønberg BH, Marosi C, et al. Temozolomide versus standard 6-week radiotherapy versus hypofractionated radiotherapy in patients older than 60 years with glioblastoma: the Nordic randomised, phase 3 trial. Lancet Oncol. 2012 Sep;13(9):916-26.
10. NCT00002840 (EORTC 26951): van den Bent MJ, Brandes AA, Taphoorn MJ, at al. Adjuvant procarbazine, lomustine, and vincristine chemotherapy in newly diagnosed anaplastic oligodendroglioma: long-term follow-up of EORTC brain tumor group study 26951. J Clin Oncol. 2013 Jan 20;31(3):344-50.
11. NCT01502241 (NOA-08): Wick W, Platten M, Meisner C, et al.; NOA-08 Study Group of Neuro-oncology Working Group (NOA) of German Cancer Society. Temozolomide chemotherapy alone versus radiotherapy alone for malignant astrocytoma in the elderly: the NOA-08 randomised, phase 3 trial. Lancet Oncol. 2012 Jul;13(7):707-15.
12. NCT00182819 (EORTC 22033-26033): Baumert BG, Hegi ME, van den Bent MJ, et al. Temozolomide chemotherapy versus radiotherapy in high-risk low-grade glioma (EORTC 22033-26033): a randomised, open-label, phase 3 intergroup study. Lancet Oncol. 2016 Nov;17(11):1521-1532.
13. NCT00006353 (EORTC 22981-26981 (Stupp)): Stupp R, Mason WP, van den Bent MJ, et al; European Organisation for Research and Treatment of Cancer Brain Tumor and Radiotherapy Groups; National Cancer Institute of Canada Clinical Trials Group. Radiotherapy plus concomitant and adjuvant temozolomide for glioblastoma. N Engl J Med. 2005 Mar 10;352(10):987-96.
14. NCT01290939 (EORTC 26101): Wick W, Gorlia T, Bendszus M, et al. Lomustine and Bevacizumab in Progressive Glioblastoma. N Engl J Med. 2017 Nov 16;377(20):1954-1963.
15. NCT00626990 (EORTC 26053-22054 (CATNON)): van den Bent MJ, Tesileanu CMS, Wick W, et al. Adjuvant and concurrent temozolomide for 1p/19q non-co-deleted anaplastic glioma (CATNON; EORTC study 26053-22054): second interim analysis of a randomised, open-label, phase 3 study. Lancet Oncol. 2021 Jun;22(6):813-823.
16. NCT03345095 (EORTC 1709 / CCTG CE.8 (MIRAGE)): Roth, Patrick et al. “Marizomib for patients with newly diagnosed glioblastoma: A randomized phase 3 trial.” *Neuro-oncology* vol. 26,9 (2024): 1670-1682. doi:10.1093/neuonc/noae053

Supplementary Table S1: Mean baseline scores of selected EORTC QLQ-C30 scales in 4301 patients with glioma versus the general population (normative or reference data).

|  | Variables/ HRQoL Scales | Physical Functioning | | | Social Functioning | | Role Functioning | |
| --- | --- | --- | --- | --- | --- | --- | --- | --- |
| ALL PATIENTS | Total patients  Glioma data mean (95% CI)  Norm data mean (sd)  Mean difference | 81.7 (80.3 to 83.0)  85.1 (18.9)  -3.4 (-4.8 to -2.1) | | | 72.0 (70.2 to 73.8)  86.2 (24.1)  -14.2 (-16.0 to -12.4) | | 68.5 (66.5 to 70.5)  84.3 (24.6)  -15.8 (-17.7 to -13.8) | |
| SEX | **Groups** | **Women** | | **Men** | **Women** | **Men** | **Women** | **Men** |
|  | Sex Glioma data mean (95% CI)  Norm data mean (sd)  Mean difference | 78.3 (76.1 to 80.5)  84.3 (18.5)  -6.0 (-8.3 to -3.8) | | 83.8 (82.1 to 85.3)  86.0 (19.3)  -2.2 (-3.9 to -0.6) | 69.8 (66.8 to 72.8)  85.7 (24.6)  -15.9 (-18.9 to -12.8) | 73.3 (71.0 to 75.5)  86.7 (23.6)  -13.4 (-15.7 to -11.1) | 66.5 (63.3 to 69.7)  84.1 (24.6)  -17.6 (-20.8 to -14.3) | 69.8 (67.3 to 72.3)  84.5 (24.5)  -14.7 (-17.3 to -12.2) |
| AGE-GROUP | Age-group 18-29 years  Glioma data mean (95% CI)  Norm data mean (sd)  Mean difference | 81.0 (68.7 to 91.8) 88.9 (14.5)  -7.9 (-19.7 to 2.9) | | 89.2 (82.6 to 94.9)  85.6 (21.6)  3.6 (-2.8 to 9.3) | 62.8 (42.3 to 82.1)  86.1 (24.7)  -23.2 (-43.8 to -4.0) | 81.4 (70.5 to 91.0)  84.4 (26.6)  -3.0 (-13.9 to 6.6) | 82.1 (66.7 to 94.9)  89.1 (20.2)  -7.1 (-22.4 to 5.8) | 74.4 (60.3 to 87.2)  82.5 (26.3)  -8.2 (-22.3 to 4.7) |
|  | Age-group 30-39 years  Glioma data mean (95% CI)  Norm data mean (sd)  Mean difference | 82.22 (75.2 to 88.3) 86.7 (18.0)  -4.4 (-11.1 to 1.6) | | 87.2 (82.0 to 91.8)  87.3 (19.0)  -0.1 (-5.2 to 4.6) | 76.4 (67.1 to 85.2)  83.7 (26.4)  -7.3 (-16.6 to 1.5) | 78.9 (72.8 to 85.0)  84.8 (25.2)  -5.8 (-12.3 to 0.2) | 72.2 (61.6 to 82.4)  84.6 (24.6)  -12.4 (-23.5 to -2.2) | 75.5 (68.1 to 82.4)  85.2 (23.4)  -9.7 (-17.1 to -2.6) |
|  | Age-group 40-49 years  Glioma data mean (95% CI)  Norm data mean (sd)  Mean difference | 83.2 (78.9 to 87.0) 85.8 (18.8)  -2.6 (-6.8 to 1.4) | | 85.9 (82.2 to 89.2)  87.9 (18.0)  -2.0 (-5.8 to 1.3) | 63.9 (57.7 to 70.1)  83.2 (25.7)  -19.3 (-25.7 to -13.1) | 71.9 (66.8 to 76.8)  85.3 (24.1)  -13.4 (-18.5 to -8.5 ) | 67.2 (60.4 to 73.8)  84.1 (25.1)  -16.9 (-23.5 to -10.7) | 70.4 (64.9 to 75.8)  85.3 (24.5)  -14.8 (-20.5 to -9.4) |
|  | Age-group 50-59 years  Glioma data mean (95% CI)  Norm data mean (sd)  Mean difference | 76.8 (72.3 to 80.9) 83.4 (18.8)  -6.6 (-11.0 to -2.6) | | 83.7 (81.0 to 86.3)  86.8 (18.2)  -3.1 (-5.8 to -0.5) | 70.7 (64.8 to 76.4)  83.8 (25.7)  -13.1 (-19.0 to -7.5) | 71.1 (67.4 to 74.9)  87.6 (22.1)  -16.4 (-20.2 to -12.7) | 65.3 (59.3 to 71.2)  82.3 (25.5)  -17.0 (-22.9 to -11.1) | 66.9 (62.5 to 71.0)  84.3 (25.1)  -17.3 (-21.7 to -13.9) |
|  | Age-group 60-69 years  Glioma data mean (95% CI)  Norm data mean (sd)  Mean difference | 74.2 (69.5 to 78.7) 82.1 (18.7)  -7.9 (-12.5 to -3.4) | | 79.1 (75.3 to 82.7)  84.9 (18.3)  -5.9 (-9.7 to -2.2) | 73.0 (67.7 to 78.4)  88.1 (22.7)  -15.0 (-20.2 to -9.7) | 73.6 (68.7 to 78.4)  89.8 (20.8)  -16.2 (-21.2 to -11.4) | 63.9 (57.9 to 70.1)  83.5 (25.3)  -19.6 (-25.9 to -13.4) | 67.5 (62.1 to 72.7)  85.4 (23.5)  -18.0 (-23.5 to -12.5) |
|  | Age-group ≥70 years  Glioma data mean (95% CI)  Norm data mean (sd)  Mean difference | 75.6 (66.0 to 83.5) 78.5 (19.8)  -2.9 (-12.2 to 5.1) | | 85.1 (79.7 to 89.8)  82.7 (19.6)  2.4 (-2.9 to 7.2) | 69.6 (56.5 to 81.9)  89.0 (20.4)  -19.4 (-32.5 to -7.1) | 72.1 (63.5 to 80.6)  90.2 (19.3)  -18.1 (-26.7 to -10.0) | 62.3 (49.3 to 74.6)  80.7 (26.4)  -18.4 (-31.4 to -6.1) | 77.5 (69.4 to 85.1)  84.8 (22.7)  -7.3 (-15.4 to 0.3) |
|  | | | | | | | | |
|  | **Variables/HRQoL Scales** | | **Cognitive Functioning** | | **Fatigue** | | **Pain** | |
| ALL PATIENTS | Total patients  Glioma data mean (95% CI)  Norm data mean (sd)  Mean difference | | 69.9 (68.1 to 71.6)  84.6 (21.3)  -14.7 (-16.5 to -13.0) | | 33.3 (31.7 to 34.9)  29.5 (25.5)  3.8 (2.3 to 5.4) | | 13.0 (11.6 to 14.4)  23.5 (27.1)  -10.5 (-11.9 to -9.1) | |
| SEX | **Groups** | **Women** | | **Men** | **Women** | **Men** | **Women** | **Men** |
|  | Sex  Glioma data mean (95% CI)  Norm data mean (sd)  Mean difference | 68.3 (65.5 to 71.0)  84.3 (20.9)  -16.0 (-18.9 to -13.2) | | 70.8 (68.6 to 72.9)  85.2 (21.7)  -14.4 (-16.6 to -12.2) | 37.0 (34.4 to 39.7)  31.7 (25.9)  5.3 (2.6 to 8.0) | 31.0 (29.1 to 32.9)  27.1 (24.8)  3.9 (2.0 to 5.8) | 15.9 (13.3 to 18.5)  25.3 (27.9)  -9.4 (-11.9 to -6.8) | 11.2 (9.6 to 12.9)  21.6 (26.0)  -10.4 (-12.0 to -8.8) |
| AGE-GROUP  AGE-GROUP | Age-group 18-29 years  Glioma data mean (95% CI)  Norm data mean (sd)  Mean difference | 79.5 (65.4 to 91.0) 82.8 (22.4)  -3.3 (-17.4 to 9.5) | | 84.0 (75.0 to 91.7)  81.3 (27.5)  2.7 (-6.3 to 10.4) | 38.5 (26.5 to 50.4)  34.4 (25.3)  4.0 (-7.0 to 15.2) | 25.2 (16.7 to 35.0)  30.7 (25.2)  -5.5 (-14.0 to 3.9) | 21.8 (11.5 to 33.3)  20.6 (24.9)  1.1 (-9.1 to 12.7) | 7.7 (2.6 to 14.1)  21.3 (26.6)  -13.6 (-18.7 to -7.2) |
|  | Age-group 30-39 years  Glioma data mean (95% CI)  Norm data mean (sd)  Mean difference | 70.4 (61.1 to 78.7)  82.9 (23.5)  -12.5 (-21.8 to -4.2) | | 77.0 (71.1 to 82.4)  84.5 (23.3)  -7.5 (-13.4 to -2.1) | 40.1 (31.2 to 49.7)  34.6 (27.3)  5.5 (-3.7 to 14.8) | 30.1 (24.7 to 35.6)  29.6 (25.0)  0.4 (-4.9 to 6.0) | 23.6 (13.4 to 35.2)  23.3 (27.7)  0.3 (-9.9 to 11.4) | 12.0 (7.1 to 17.9)  22.1 (25.9)  -10.1 (-15.0 to -4.5) |
|  | Age-group 40-49 years  Glioma data mean (95% CI)  Norm data mean (sd)  Mean difference | 68.5 (62.5 to 74.4) 82.7 (22.7)  -14.2 (-20.2 to -8.3) | | 74.2 (69.4 to 78.8)  85.7 (20.7)  -11.5 (-16.3 to -6.9) | 36.3 (31.1 to 41.6)  33.9 (27.1)  2.4 (-2.8 to 7.6) | 30.4 (26.1 to 34.6)  27.5 (24.5)  2.9 (-1.3 to 7.1) | 17.0 (12.3 to 22.0)  25.2 (28.1)  -8.2 (-12.7 to -3.2) | 12.5 (9.0 to 16.1)  21.4 (26.05)  -8.9 (-12.3 to -5.3) |
|  | Age-group 50-59 years  Glioma data mean (95% CI)  Norm data mean (sd)  Mean difference | 68.2 (63.0 to 73.4) 83.2 (21.1)  -15.0 (-20.2 to -10.0) | | 67.5 (63.7 to 71.3) 86.4 (20.8)  -18.8 (-22.7 to -15.1) | 35.6 (30.9 to 40.4)  32.1 (26.2)  3.6 (-1.1 to 8.4) | 34.0 (30.6 to 37.3)  26.7 (25.1)  7.2 (3.9 to 10.6) | 13.1 (9.4 to 17.2)  28.7 (29.2)  -15.5 (-19.4 to -11.4) | 12.6 (9.8 to 15.6)  22.9 (26.3)  -10.3 (-13.1 to -7.3) |
|  | Age-group 60-69  Glioma data mean (95% CI)  Norm data mean (sd)  Mean difference | 65.8 (60.3 to 71.1) 87.9 (16.6)  -22.0 (-27.4 to -16.8) | | 68.0 (63.2 to 72.5)  87.9 (16.6)  -20.0 (-24.7 to -15.5) | 36.2 (30.9 to 41.6)  26.6 (24.5)  9.5 (4.2 to 14.8) | 29.7 (25.7 to 33.9)  23.5 (23.9)  6.3 (2.2 to 10.5) | 12.5 (8.1 to 17.7)  25.4 (28.6)  -12.9 (-17.5 to -7.9) | 8.2 (5.4 to 11.4)  22.1 (26.0)  -13.9 (-16.7 to -10.7) |
|  | Age-group ≥70 years  Glioma data mean (95% CI)  Norm data mean (sd)  Mean difference | 68.8 (58.0 to 79.0) 86.6 (17.2)  -17.7 (-28.6 to -7.6) | | 67.6 (58.6 to 76.1)  87.7 (14.9)  -20.1 (-29.6 to -11.6) | 44.0 (33.3 to 55.1)  28.1 (24.2)  15.9 (4.7 to 27.0) | 27.9 (21.9 to 34.2)  21.9 (23.3)  6.0 (0.0 to 12.0) | 21.7 (10.1 to 34.8)  28.8 (28.2)  -7.0 (-18.7 to 6.0) | 11.7 (6.3 to 18.0)  19.7 (25.0)  -8.0 (-13.8 to -1.7) |

Abbreviations: SD: standard deviation, CI: confidence interval

Dark highlighted cells represent groups with both statistically significant and clinically relevant changes.
