# Supplementary figures and images for: Age and sex as risk factors for health-related quality of life outcomes in patients with glioma: a CODAGLIO 2.0 analysis
Source: Oncologist. 2026 Jan 22;31(3):oyag005. doi: 10.1093/oncolo/oyag005 (PMC12948937; doi:10.1093/oncolo/oyag005)

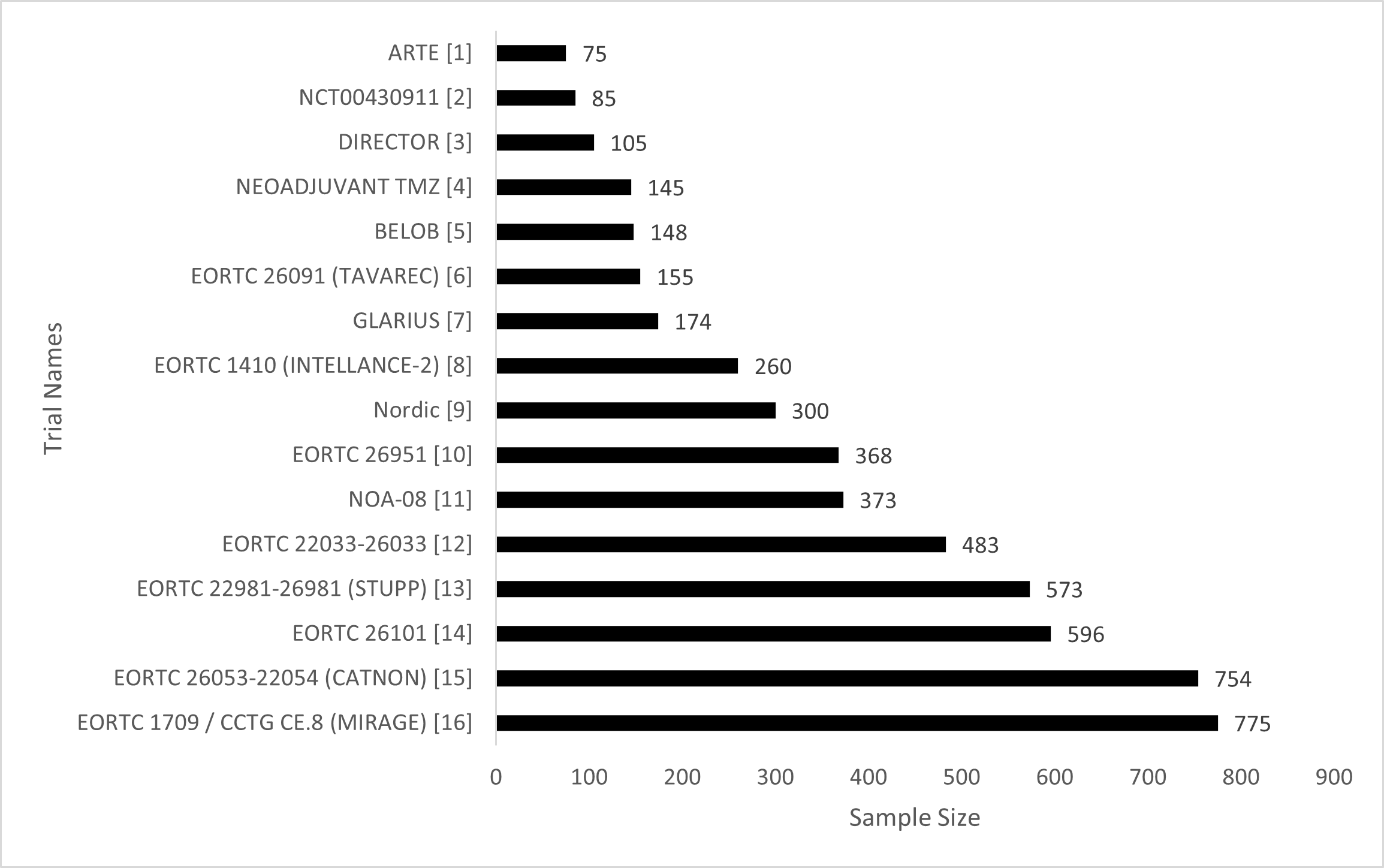

Supplement: oyag005_Supplementary_Data [file oyag005_supplementary_data.zip › Supplementary File 1a 16 closed phase (1).tif]
